# Supplementary material for: The Antimicrobial Resistance (AMR) Rates of Enterobacterales in a Rural Hospital from the Eastern Region, Ghana: A Retrospective Study, 2022
Source: Antibiotics (Basel). 2023 Aug 16;12(8):1321. doi: 10.3390/antibiotics12081321 (PMC10451727; doi:10.3390/antibiotics12081321)
Supplement: Supplementary file 1 [file antibiotics-12-01321-s001.zip › antibiotics-2540864-supplementary.pdf]

Supplementary Material

Table S1. Biochemical tests for microorganism identification performed on specific agar, including urea, citrate, indole, and Triple Sugar Iron (TSI) tests. The combinations shown led to the identification of a specific bacterial genus and species. H_2S: Hydrogen sulfide gas (Blackening). D: Different strains give different reactions. Y: Yellow colour. R: Red colour.

				**TSI MEDIUM**			
**SPECIES**	**Urea**	**Citrate**	**Indole**	**Slope**	**Butt**	**H_2S**	**Gas**
Escherichia Coli	-	-	+2	Y6	Y	-	+2
Shigella spp.	-	-	d	R	Y	-	-3
Salmonella typhi	-	-	-	R	Y	+	-
Salmonella paratyphi A	-	-	-	R	Y	-	+
Most other Salmonellae	-	+	-	R	Y	+2	D
Citrobacter freundii	D	+	-3	R or Y	Y	D	+
Klebsiella pneumonia	+slow	+2	-3	Y	Y	-	+
Klebsiella oxytoca	+slow	+2	+	Y	Y	-	+
Enterobacter spp.	-	+	-	Y	Y	-	+
Serratia marcenses	D	+	-	R or Y	Y	-	D
Proteus vulgaris	+	D	+	R	Y	+	D
Proteus mirabilis	+	+2	-	R	Y	+	+
Morganella morganii	+	-	+	R	Y	-	D
Providencia spp.	D	+	+	R	Y	-	D
Yersinia enterocolitica	+slow	-	D	R	Y	-	-
Vibrio cholera	-	D	+	R	Y	-	-
Vibrio parahaemolyticus	-3	D	+	R	Y	-	-
